# Eldecalcitol and calcium supplementation in Chinese women with postmenopausal osteoporosis: a prospective observational cohort study

**DOI:** 10.1007/s00774-026-01735-x

**Published:** 2026-06-16

**Authors:** Yan Jiang, Yuhong Zeng, Li Xin, Wei Chen, Youjia Xu, Shoujin Tian, Qinglai Wang, Jianjun Li, Shihong Chen, Xuanxia Lin, Qifeng Ying, Shun Chiba, Chiemi Yamagiwa, Tomohiro Yamaguchi, Zheng Ke, Weibo Xia, Yasuhiro Takeuchi

**Affiliations:** 1 Peking Union Medical College Hospital, No.1 Shuai-Fu-Yuan, Beijing, 100730 China; 2 Xi’an Hong Hui Hospital, No.555 East Youyi Road Nanshao Gate anguo Road No.76, Xi’an, 710054 Shanxi China; 3 The Affiliated Hospital of Xuzhou Medical University, No.99 West Huaihai Road, Xuzhou, 221006 Jiangsu China; 4 Xinyi Hospital of Traditional Chinese Medicine, No.168 Daqiao Dong Lu, Xinyi, 221416 Jiangsu China; 5 The Second Affiliated Hospital of Soochow University, No.1055 Sanxiang Road Gusu District, Suzhou, 215004 Jiangsu China; 6 Zhangjiagang First People’s Hospital, No.68, Jiyang West Road, Zhangjiagang, 215638 Jiangsu China; 7 Wenzhou TCM Hospital of Zhejiang Chinese Medical University, No.9, Jiaowei Road, Liuhongqiao Nanjiao Street, Lucheng District, Wenzhou, 325088 Zhejiang China; 8 First People’s Hospital of Jinan, No.132 Daminghu Road, Lixia District, Jinan, 250011 Shandong China; 9 The Second Hospital of Shandong University, 247 Beiyuan Dajie, Jinan, 250011 Shandong China; 10 ZhongShan Chen Xinghai Hospital of Integrated Traditional Chinese and Western Medicine, No.18 Zhuyuan Road, Xiaolan Town, Zhongshan, 314408 Guangdong China; 11 Zhejiang Provincial People’s Hospital, No.158 Shangtang Road Gongshu District, Hangzhou, 528455 Zhejiang China; 12 Safety Science 2 Department, Drug Safety Division, Chugai Pharmaceutical Co., Ltd., 1‑1 Nihonbashi‑Muromachi 2-Chome, Chuo-ku, Tokyo 103‑8324 Japan; 13 Safety Science 1 Department, Drug Safety Division, Chugai Pharmaceutical Co., Ltd., 1‑1 Nihonbashi‑Muromachi 2-Chome, Chuo-ku, Tokyo 103‑8324 Japan; 14 Medical Division, Chugai Pharma China Co., Ltd., Unit 5702, Hong Kong New World Tower, No.300, Central Huai Hai Road, Huangpu District, Shanghai, 200021 China; 15 Toranomon Hospital Kajigaya, 1-3-1 Kajigaya, Takatsu-ku, Kawasaki, Kanagawa 213-8587 Japan; 16 Okinaka Memorial Institute for Medical Research, 2‑2‑2 Toranomon, Minato‑ku, Tokyo 105‑8470 Japan

**Keywords:** Eldecalcitol, Hypercalcemia, Adverse drug reactions, Osteoporosis

## Abstract

**Introduction:**

Eldecalcitol is an active vitamin D_3_ derivative approved for the treatment of osteoporosis in Japan and postmenopausal osteoporosis in China. However, the impact of concomitant calcium supplementation on its safety profile is unclear.

**Materials and methods:**

This was a prospective, observational, non-interventional post-marketing drug intensive monitoring study in Chinese postmenopausal women with osteoporosis (NCT05433207). Data were collected using an electronic case report form at baseline, before initial eldecalcitol dosing, and during routine clinical visits over a 1-year follow-up period.

**Results:**

One thousand patients were enrolled from 29 Chinese clinical sites; 958 received eldecalcitol (853 without/105 with calcium supplementation). The mean age (standard deviation [SD]) of patients was 65.6 (9.0) years and mean (SD) time since osteoporosis diagnosis was 2.7 (3.8) years. Adverse drug reactions (ADRs) affected 39.2% and 42.9% of patients in the without and with calcium supplementation groups, respectively. 22 (2.3%) patients developed hypercalcemia including 17 (1.99%) and 5 (4.76%) in the without and with calcium supplementation groups, respectively. Multivariable logistic regression analyses indicated that those receiving calcium were more likely to experience hypercalcemia (adjusted odds ratio [OR] 3.07, 95% confidence interval [CI] 1.16, 8.10, *P* = 0.02) than those not receiving calcium. ADRs of urolithiasis were experienced by three patients (0.35%) in the without calcium supplementation group.

**Conclusion:**

This study confirmed the real-world safety profile of eldecalcitol in a large population of Chinese women with postmenopausal osteoporosis. Hypercalcemia incidence was higher in patients with compared to without calcium supplementation, indicating that concomitant eldecalcitol and calcium should be carefully considered.

**Supplementary Information:**

The online version contains supplementary material available at https://doi.org/10.1007/s00774-026-01735-x.

## Introduction

Osteoporosis is a common skeletal disease characterized by low bone mass and microstructural damage to bone tissue [1], which can lead to bone pain and increased risk of fracture [2]. A 2021 systematic review and meta-analysis reported an osteoporosis global prevalence of 23.1% in women as compared with 11.7% in men [3]. In 2022, a meta-analysis showed the prevalence of osteoporosis in postmenopausal women in China was estimated at 38.8% [4]. Postmenopausal women are particularly vulnerable to osteoporosis due to the rapid decline in estrogen levels after menopause [5, 6] leading to accelerated bone resorption, which outpaces bone formation and is associated with bone demineralization [6, 7].

Eldecalcitol is a synthesized active vitamin D₃ [1,25(OH)₂D₃] derivative used to treat osteoporosis [8]. Compared with another vitamin D analog, alfacalcidol, eldecalcitol decreased the relative risk of vertebral fractures by 26.0% in a randomized, double-blind study over 3 years [8]. The efficacy and safety of eldecalcitol were also verified in Chinese osteoporotic patients [9]. Eldecalcitol was first approved in Japan in 2011 and was approved for the treatment of postmenopausal osteoporosis in China in 2020. The improved calcium (Ca) absorption associated with eldecalcitol versus alfacalcidol may, in part, reflect eldecalcitol’s greater affinity for serum vitamin D-binding protein, which extends its plasma half-life [10]. Therefore, the impact of eldecalcitol therapy on calcium metabolism should be considered. A Japanese post-marketing surveillance study reported hypercalcemia (0.94%) in women [11]. A real-world study in Japanese patients with osteoporosis also confirmed the long-term safety of eldecalcitol [11, 12].

Since Ca may be used concomitantly in anti-osteoporotic treatment in real-world settings, concomitant use of Ca with eldecalcitol would potentially increase the incidence of hypercalcemia [9]. Therefore, this observational, prospective cohort study aimed to compare the incidence of hypercalcemia in postmenopausal women with osteoporosis receiving eldecalcitol with Ca and without Ca supplementation, and to monitor the type, incidence, severity, and relevance of other adverse drug reactions (ADRs), including urolithiasis.

## Materials and methods

### Study design and setting

This real-world, multicenter, prospective, observational, non-interventional cohort study was conducted for post-marketing surveillance of eldecalcitol in Chinese postmenopausal women with osteoporosis (NCT05433207). The study included qualified medical institutions across various Chinese provinces. One thousand patients were enrolled and followed for 1 year. The first patient was enrolled in January 2023 and the last patient completed the study in September 2024.

Physicians were responsible for monitoring patients and collecting safety information using customized electronic case report forms during clinical visits, including a baseline visit with the first dosing with eldecalcitol and 4 follow-up visits. Physicians recorded the time, dosage, and specifications; collected data on patient laboratory tests, clinical diagnosis, Ca supplements, other medications, and adverse events (AEs). Data collection occurred during 2 phases: the baseline phase, which commenced 1.5 months prior to the first dose of eldecalcitol, and the follow-up phase, which extended for 1 year from the first eldecalcitol dose, with measurements collected at 3-, 6-, 9-, and 12-month visits. Medical history of urolithiasis was collected at baseline and during the follow-up phase. There were no mandatory requirements regarding baseline imaging assessments such as computed tomography or ultrasound, nor were patients required to visit the hospital specifically for surveillance purposes, as the study was conducted under real-world clinical practice conditions. The follow-up period extended from the time of informed consent until eldecalcitol cessation, patient transfer or discontinuation of visits, or the last day of data collection; patients who stopped eldecalcitol due to an AE were additionally followed until AE outcome. Patients with no visits or laboratory tests within the year after their first treatment with eldecalcitol were considered lost to follow-up.

### Ethical approval

Independent Ethics Committees from each clinical site provided written approval of the study protocol and related documents. Patients provided written informed consent before participating in the study.

### Patients

The study included postmenopausal Chinese women diagnosed with osteoporosis, who were prescribed eldecalcitol by their physician according to the approved dose of 0.75 μg, once a day and with a permitted dose reduction to 0.5 μg, once a day. Patients were eligible for enrollment if they provided a signed informed consent form (ICF), were prescribed eldecalcitol by their physician during a routine clinical visit and were expected to initiate treatment within 1.5 months of study enrollment. Any patients with contraindications to eldecalcitol were excluded.

### Sample size

The sample size was based on the finding from the Chinese clinical trial in which the incidence of hypercalcemia (serum Ca [sCa] > 10.4 mg/dL) was 5.9% following eldecalcitol treatment without Ca [9]. It was assumed that 20.0% of patients in China would receive Ca in real-world clinical practice, and that the incidence of sCa > 10.4 mg/dL in that subgroup would be 11.8%, twice the rate compared with those without Ca. Based on these results, the estimated odds ratio (OR) and its 95% CI for developing hypercalcemia in a group of 750 patients would be 2.13 (1.17–3.89). Therefore, a target sample size of 1,000 patients was selected to allow for the evaluation of the risk associated with high Ca intake, considering potential dropouts and missing data.

### Endpoints

The primary objective of the study was to compare the incidence of hypercalcemia in patients with Ca and without Ca supplementation. Hypercalcemia was defined as AEs reported by physicians that were coded as Medical Dictionary for Regulatory Activities (MedDRA) Preferred Term (PT) of “hypercalcemia” or “blood calcium increased”. The exposure variable was Ca use status (including dietary supplements) at baseline (on start date of study drug). Concomitant medication use, including calcium supplementation, was quantified during eldecalcitol treatment.

Secondary objectives included the type, incidence, severity, and relevance of other treatment-emergent adverse events (TEAEs), ADRs, serious adverse events (SAEs), and serious adverse drug reactions (SADRs). In addition, the study examined the incidence of urolithiasis, which was defined as AEs coded using MedDRA PTs of “ureterolithiasis”, “nephrolithiasis”, or “urinary calculus”. Baseline characteristics included patient characteristics, osteoporosis parameters, medical history and concurrent illnesses, treatment status, and laboratory tests (sCa, albumin, serum creatinine [sCr], and urinary Ca [uCa] tests performed within 1.5 months before the first dose of eldecalcitol). Laboratory testing was repeated every 3 months (± 1.5 months) during the follow-up period.

### Statistical analysis

All analyses were performed using SAS Software version 9.4. The primary outcome and safety analyses were conducted on the safety analysis set (SAS). The SAS included all enrolled patients who had signed the ICF, received study drug at least once, and had recorded safety data. Continuous variables were summarized, including mean and standard deviation (SD). Categorical variables were summarized using counts and percentages. Event frequencies were provided where appropriate. Missing data were reported but not imputed unless otherwise specified.

Safety outcome summaries and events leading to discontinuation or death were summarized using frequencies (counts and percentages) and 95% CIs, by Ca use status and overall. For the exploratory analysis, hypercalcemia events were summarized by Ca use status and overall, by explanatory variables. Drug-related events were summarized overall and by action taken with the study drug, outcome, and Ca use status.

An analysis was conducted based on patient’s status of Ca use at baseline and the incidence of hypercalcemia. This analysis was performed using univariate and multivariable logistic regression models, OR with 95% CIs and *P*-values were reported. A stepwise selection procedure was used to identify potential prognostic variables to control for confounding, including baseline Ca use (yes or no), age category (< 65 or ≥ 65 years), body mass index (BMI) category (< 25.0 or ≥ 25.0 kg/m^2^ or unknown), fragility fractures (yes, no, or unknown), previous therapeutic medications for osteoporosis (yes or no), baseline-adjusted sCa category (≤ 10.4 or > 10.4 mg/dL), baseline estimated glomerular filtration rate (eGFR) category (< 60.0 or ≥ 60.0 mL/min/1.73 m^2^), status of parathyroid hormone use (yes or no), primary hyperparathyroidism (yes or no), and malignancy (yes or no). A Firth logistic model with penalized likelihood estimation was applied when the standard logistic analysis was unstable due to a zero event for the outcome of interest. This model used covariates previously identified through a stepwise selection procedure in the general multivariable logistic model.

In a post hoc analysis, statistical comparisons were performed between patients without and with Ca supplementation for demographics and baseline characteristics, laboratory testing, concomitant medications, and the summary of adverse events. Welch's t-test for continuous variables and chi-square test for categorical variables were used. Additionally, clinical characteristics including baseline-adjusted sCa, baseline eGFR, and concomitant medications were compared between patients who developed hypercalcemia and those who did not using univariate logistic regression analyses, reporting ORs with 95% CIs and *P*-values. Anti-RANKL therapy (denosumab) use was also evaluated as a candidate explanatory variable in the multivariable logistic regression analysis with stepwise variable selection. Furthermore, a sensitivity analysis excluding patients with primary hyperparathyroidism was conducted to assess the impact of Ca co-administration on hypercalcemia using multivariable logistic regression analysis.

## Results

### Patient disposition and baseline characteristics

**Fig. 1:**
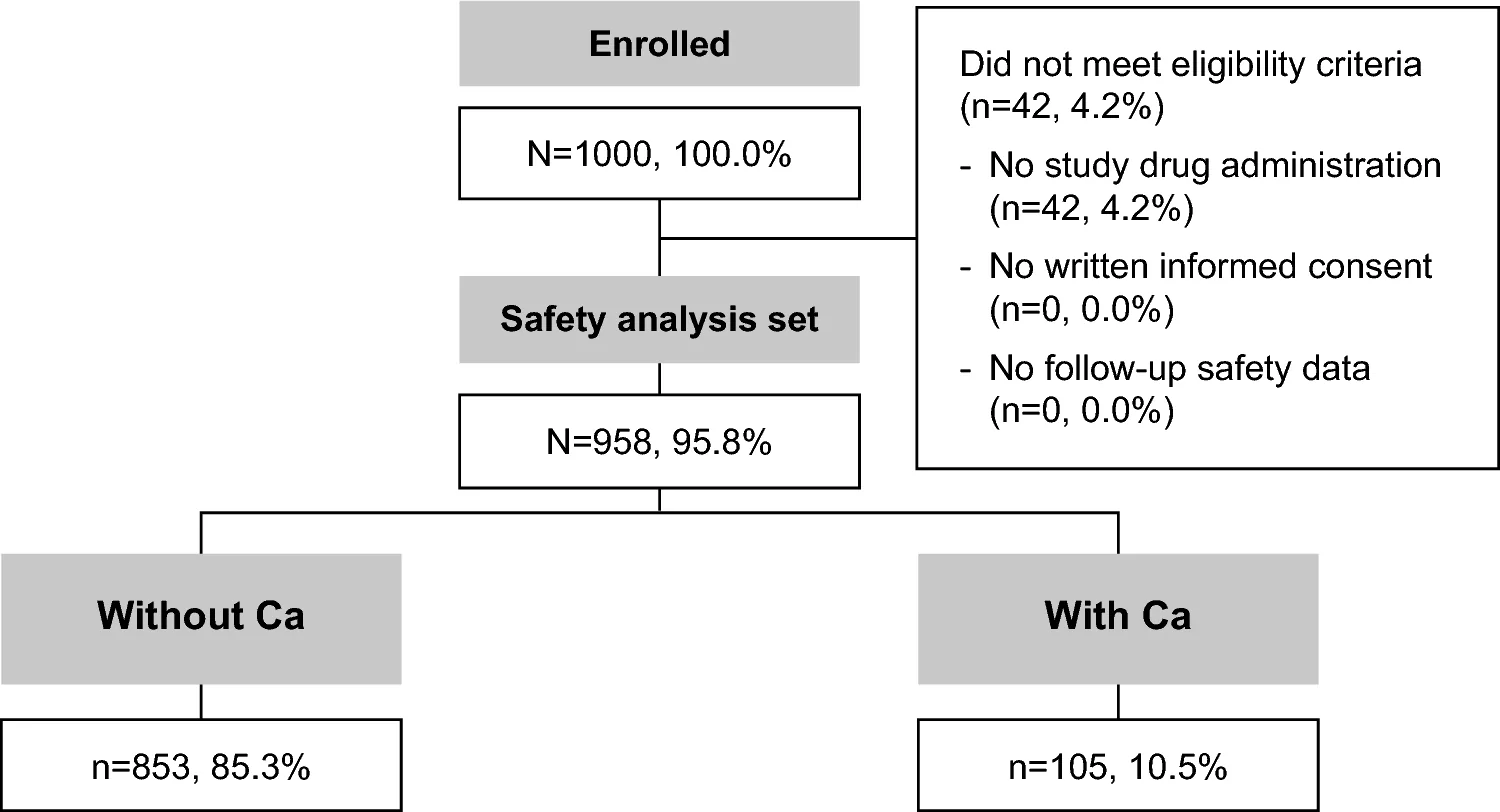
Patient disposition. The safety analysis set (*SAS*) included all enrolled patients who signed the informed consent, received at least one dose of study drug and had recorded safety data. *Ca* calcium

Of the 1,000 Chinese women with postmenopausal osteoporosis enrolled from 29 clinical sites across China, 42 did not receive study drug, and 958 patients were included in the SAS (Fig. 1). Of those, 105 and 853 were included in the with Ca and without Ca groups based on the status of Ca use at baseline (on start of study drug), respectively.

In the overall population, the mean (SD) age and BMI were 65.6 (9.0) years and 23.6 (3.4) kg/m^2^, respectively. Of the 958 patients, the mean (SD) time since diagnosis of osteoporosis was 2.7 (3.8) years and 241 patients (25.16%) had a history of fragility fractures (Table 1). Nineteen patients (1.98%) had urolithiasis, 10 patients (1.04%) had primary hyperparathyroidism, 34 patients (3.55%) had malignancy. One patient with Graves’ disease was identified in the without Ca group. Two patients with primary adrenal insufficiency were reported, one in the with Ca group and one in the without Ca group. No cases with a history of lithium use were reported. The baseline-adjusted sCa levels in the patients with primary hyperparathyroidism, Graves' disease, and adrenal insufficiency were within the normal range (8.68–10.32 mg/dL). In addition, no cases of hypercalcemia were reported as a comorbidity in these patients (Table 1).

**Table 1:** Demographics and baseline characteristics by completion status and analysis sets

Parameter	Overall population N = 958	Without Ca N = 853	With Ca N = 105	*P*-value
Age (years), mean ± SD	65.64 ± 9.03	65.64 ± 8.87	65.64 ± 10.33	0.9985
Chinese ethnicity, n (%)				0.4603
Han	952 (99.37)	848 (99.41)	104 (99.05)	
Mongolian	2 (0.21)	2 (0.23)	0 (0.00)	
Hui	1 (0.10)	1 (0.12)	0 (0.00)	
Zhuang	1 (0.10)	1 (0.12)	0 (0.00)	
Manchu	2 (0.21)	1 (0.12)	1 (0.95)	
Characteristics, mean ± SD				
Height (cm)	157.24 ± 5.80 (n = 949)	157.25 ± 5.83 (n = 848)	157.21 ± 5.65(n = 101)	0.9526
Weight (kg)	58.24 ± 8.89 (n = 949)	58.33 ± 8.96 (n = 848)	57.51 ± 8.27 (n = 101)	0.3500
BMI (kg/m^2^)	23.55 ± 3.39 (n = 949)	23.59 ± 3.42 (n = 848)	23.27 ± 3.16 (n = 101)	0.3363
Information on osteoporosis				
Time since diagnosis^a^, mean ± SD	2.67 ± 3.80 (n = 956)	2.68 ± 3.73 (n = 852)	2.61 ± 4.35 (n = 104)	0.8630
History of fragility fractures, n (%)	241 (25.16)	214 (25.09)	27 (25.71)	0.8890
Medical history, n (%)				
Renal impairment	35 (3.65)	25 (2.93)	10 (9.52)	0.0007
Hepatic impairment	78 (8.14)	66 (7.74)	12 (11.43)	0.1919
Malignancy	34 (3.55)	30 (3.52)	4 (3.81)	0.8785
Primary hyperparathyroidism	10 (1.04)	9 (1.06)	1 (0.95)	0.9222
Urolithiasis	19 (1.98)	17 (1.99)	2 (1.90)	0.9512
Diabetes mellitus	242 (25.26)	201 (23.56)	41 (39.05)	0.0006
Dyslipidemia	123 (12.84)	98 (11.49)	25 (23.81)	0.0004
Chronic gastritis	92 (9.60)	83 (9.73)	9 (8.57)	0.7037
Intervertebral disc protrusion	81 (8.46)	67 (7.85)	14 (13.33)	0.0569
Arteriosclerosis	46 (4.80)	34 (3.99)	12 (11.43)	0.0008
Cerebrovascular disorder	33 (3.44)	29 (3.40)	4 (3.81)	0.8280
Myocardial ischemia	12 (1.25)	11 (1.29)	1 (0.95)	0.7694
Rheumatoid arthritis	14 (1.46)	14 (1.64)	0 (0.00)	0.1860
Clinical status, mean ± SD or n (%)				
Baseline sCa (mg/dL)	9.35 ± 0.43 (n = 806)	9.36 ± 0.42 (n = 718)	9.29 ± 0.48 (n = 88)	0.1745
Baseline-adjusted sCa (mg/dL)^b^	9.39 ± 0.41 (n = 806)	9.39 ± 0.40 (n = 718)	9.34 ± 0.43 (n = 88)	0.2889
Baseline albumin (g/dL)	4.36 ± 0.42 (n = 679)	4.37 ± 0.41 (n = 600)	4.27 ± 0.49 (n = 79)	0.0874
Baseline sCr (mg/dL)	0.66 ± 0.18 (n = 700)	0.65 ± 0.16 (n = 620)	0.75 ± 0.25 (n = 80)	0.0006
Baseline uCa (mg/day)	175.22 ± 112.34 (n = 184)	187.94 ± 116.61 (n = 143)	130.83 ± 82.90 (n = 41)	0.0007
Baseline eGFR (mL/min/1.73 m^2^)^c^	90.28 ± 15.27 (n = 700)	91.16 ± 14.29 ((n = 620)	83.49 ± 20.29 (n = 80)	0.0015

Data are mean ± SD or n (%), unless stated otherwise. Percentages calculated based on the total number of patients per cohort (N)

*BMI* body mass index, *Ca* calcium, *eGFR* estimated glomerular filtration rate, *N* the number of enrolled patients, *n* the number of patients in each category, *PTH* parathyroid hormone, *sCa* serum calcium, *sCr* serum creatinine, *SD* standard deviation, *uCa* urinary calcium

^a^Time since diagnosis = the date of the informed consent—the date of disease diagnosis/365.25

^b^If albumin was < 4 g/dL then sCa = sCa + (4—albumin concentration). If albumin was ≥ 4.0 g/dL the sCa = sCa. If baseline albumin was missing, it was imputed to 4.0 g/dL

^c^141 × minimum (sCr/0.7, 1) ^(−0.329)^ × maximum (sCr/0.7, 1) ^(−1.209)^ × 0.993 ^Age^ × 1.018

The frequencies of some medical histories were higher in the with Ca group than in the without Ca group, such as renal impairment, diabetes mellitus, dyslipidemia, and arteriosclerosis. When comparing baseline laboratory values in patients without and with Ca, sCa, albumin, and baseline-adjusted sCa values were found to be similar (Table 2A); mean** ± **SD baseline sCr (mg/dL) was higher in the with Ca group (0.75 ± 0.25) than in the without Ca group (0.65 ± 0.16), whereas baseline uCa was higher in the without Ca group than the with Ca group (187.94 ± 116.61 and 130.83 ± 82.90, respectively), as was baseline eGFR (91.16 ± 14.29 and 83.49 ± 20.29, respectively; Tables 1**, **2). To mitigate confounding for estimation of the risk of hypercalcemia on calcium supplementation, a multivariable logistic regression analysis was performed using pre-specified potential confounders (for example, eGFR) as covariates. Mean clinical status at baseline differed between the without Ca and with Ca groups with respect to sCr (0.65 and 0.75 mg/dL, respectively), uCa (187.94 and 130.83 mg/day, respectively), and eGFR (91.16 and 83.49 mL/min/1.73 m^2^, respectively (Table 1). The most common concomitant osteoporosis medications overall were vitamin D and vitamin D analogs (30.27%), denosumab (24.53%), Ca (15.14%), vitamin K2 (12.73%), and bisphosphonates (8.98%) (Table 3). Excluding calcium supplements, the medications that were used more frequently in the with Ca group were vitamin D and vitamin D analogs, as well as bisphosphonates. Conversely, vitamin K2 had a higher usage rate in the without Ca group. It should be noted that few patients received concomitant calcitriol (n = 18, 1.88%), concomitant alfacalcidol (n = 16, 1.67%), or concomitant native vitamin D (n = 71, 7.41%) while they were receiving eldecalcitol treatment. The median (min, max) overlapping days between the initiation of eldecalcitol and vitamin D or vitamin D analog administration was 120 days (1.0–476.0). Among these, the median (min, max) overlapping days for the vitamin D analogs calcitriol and alfacalcidol were 17 days (1.0–476.0) and 12 days (1.0–399.0), respectively. The median (min, max) duration of combination therapy with native vitamin D was 182 days (1.0–407.0).

**Table 2:** (A) Laboratory testing and (B) Status of Calcium use at 3, 6, 9, and 12 months

Item	Statistics	Overall population (N = 958)	Without Ca (N = 853)	With Ca (N = 105)	*P*-value
(A) Laboratory testing					
Adjusted sCa (mg/dL)					
Baseline	No. of patients (missing) Mean ± SD	806 (152) 9.39 ± 0.41	718 (135) 9.39 ± 0.40	88 (17) 9.34 ± 0.43	0.2998
Change from baseline to month 3	No. of patients (missing) Mean ± SD	445 (513) 0.05 ± 0.51	399 (454) 0.04 ± 0.51	46 (59) 0.14 ± 0.58	0.2734
Change from baseline to month 6	No. of patients (missing) Mean ± SD	386 (572) 0.07 ± 0.51	343 (510) 0.06 ± 0.51	43 (62) 0.10 ± 0.53	0.6506
Change from baseline to month 9	No. of patients (missing) Mean ± SD	275 (683) −0.06 ± 0.51	244 (609) −0.07 ± 0.51	31 (74) 0.04 ± 0.55	0.3207
Change from BASELINE to month 12	No. of patients (missing) Mean ± SD	315 (643) −0.03 ± 0.50	277 (576) −0.04 ± 0.52	38 (67) −0.01 ± 0.39	0.6686
Albumin (g/dL)					
Baseline	No. of patients (missing) Mean ± SD	679 (279) 4.36 ± 0.42	600 (253) 4.37 ± 0.41	79 (26) 4.27 ± 0.49	0.0874
Change from baseline to month 3	No. of patients (missing) Mean ± SD	288 (670) 0.06 ± 0.41	250 (603) 0.06 ± 0.40	38 (67) 0.07 ± 0.42	0.9243
Change from baseline to month 6	No. of patients (missing) Mean ± SD	303 (655) 0.10 ± 0.41	263 (590) 0.09 ± 0.40	40 (65) 0.16 ± 0.44	0.3563
Change from baseline to month 9	No. of patients (missing) Mean ± SD	208 (750) 0.04 ± 0.33	175 (678) 0.03 ± 0.34	33 (72) 0.06 ± 0.30	0.6955
Change from baseline to month 12	No. of patients (missing) Mean ± SD	238 (720) −0.05 ± 0.39	204 (649) −0.04 ± 0.40	34 (71) −0.12 ± 0.37	0.2870
sCr (mg/dL)					
Baseline	No. of patients (missing) Mean ± SD	700 (258) 0.66 ± 0.18	620 (233) 0.65 ± 0.16	80 (25) 0.75 ± 0.25	0.0006
Change from baseline to month 3	No. of patients (missing) Mean ± SD	306 (652) 0.01 ± 0.13	265 (588) 0.01 ± 0.12	41 (64) 0.01 ± 0.14	0.8703
Change from baseline to month 6	No. of patients (missing) Mean ± SD	308 (650) 0.03 ± 0.12	268 (585) 0.03 ± 0.12	40 (65) 0.01 ± 0.11	0.1633
Change from baseline to month 9	No. of patients (missing) Mean ± SD	210 (748) −0.01 ± 0.11	178 (675) −0.01 ± 0.11	32 (73) −0.01 ± 0.10	0.8365
Change from baseline to month 12	No. of patients (missing) Mean ± SD	249 (709) 0.02 ± 0.13	215 (638) 0.03 ± 0.14	34 (71) −0.03 ± 0.09	0.0019
uCa (mg/day)					
Baseline	No. of patients (missing) Mean ± SD	184 (774) 175.22 ± 112.34	143 (710) 187.94 ± 116.61	41 (64) 130.83 ± 82.90	0.0007
Change from baseline to month 3	No. of patients (missing) Mean ± SD	38 (920) 30.21 ± 176.29	26 (827) 21.38 ± 186.75	12 (93) 49.33 ± 157.13	0.6358
Change from baseline to month 6	No. of patients (missing) Mean ± SD	35 (923) −8.23 ± 113.54	19 (834) −42.53 ± 116.16	16 (89) 32.50 ± 98.83	0.0469
Change from baseline to month 9	No. of patients (missing) Mean ± SD	26 (932) −32.46 ± 116.11	16 (837) −50.25 ± 116.56	10 (95) −4.00 ± 115.50	0.3344
Change from baseline to month 12	No. of patients (missing) Mean ± SD	24 (934) −6.50 ± 111.33	11 (842) −19.64 ± 141.63	13 (92) 4.62 ± 82.14	0.6234
(B) Status of Calcium use at 3, 6, 9, and 12 months					
Time	Status of Ca use	Overall population (N = 958)	Without Ca (N = 853)	With Ca (N = 105)	
3 months		851	761	90	
	Yes	98 (11.52)	22 (2.89)	76 (84.44)	
6 months		762	687	75	
	Yes	76 (9.97)	22 (3.20)	55 (72.00)	
9 months		700	628	72	
	Yes	73 (10.43)	20 (3.18)	53 (73.61)	
12 months		649	585	64	
	Yes	60 (9.24)	15 (2.56)	46 (70.31)	

Data presented for (B) are n (%). Percentages are calculated based on the number of patients with non-missing data for each variable

*Ca* calcium, *sCa* serum calcium, *sCr* serum creatinine, *uCa* urinary calcium, *SD* standard deviation. *N* the number of patients in safety analysis set, *n* the number of patients in each category, *No*. number of patients, *missing* the number of patients with missing data for each variable

**Table 3:** Concomitant^a^ medications by preferred name

Classification medication name	Overall population (N = 958)	Without Ca (N = 853)	With Ca (N = 105)	*P*-value
**Total**	**881 (91.96)**	**776 (90.97)**	**105 (100.0)**	**0.0013**
**Calcium**	**145 (15.14)**	**40 (4.69)**	**105 (100.0)**	** < 0.0001**
Calcium carbonate; cholecalciferol	92 (9.60)	27 (3.17)	65 (61.90)	< 0.0001
Calcium carbonate	32 (3.34)	6 (0.70)	26 (24.76)	< 0.0001
Artificial tiger bone	9 (0.94)	3 (0.35)	6 (5.71)	< 0.0001
Calcium	6 (0.63)	1 (0.12)	5 (4.76)	< 0.0001
**Estrogen**	**4 (0.42)**	**3 (0.35)**	**1 (0.95)**	**0.3677**
Progesterone	2 (0.21)	2 (0.23)	0 (0.00)	0.6194
Dydrogesterone; Estradiol	1 (0.10)	0 (0.00)	1 (0.95)	0.0043
Medroxyprogesterone acetate	1 (0.10)	1 (0.12)	0 (0.00)	0.7256
**Parathyroid hormone (PTH)**	**18 (1.88)**	**17 (1.99)**	**1 (0.95)**	**0.4587**
Teriparatide	18 (1.88)	17 (1.99)	1 (0.95)	0.4587
**Calcitonin**	**50 (5.22)**	**47 (5.51)**	**3 (2.86)**	**0.2488**
Calcitonin, salmon	29 (3.03)	28 (3.28)	1 (0.95)	0.1885
Elcatonin	21 (2.19)	19 (2.23)	2 (1.90)	0.8313
**Vitamin D and vitamin D analogs**	**290 (30.27)**	**207 (24.27)**	**83 (79.05)**	** < 0.0001**
Ergocalciferol	118 (12.32)	108 (12.66)	10 (9.52)	0.3560
Calcium carbonate; cholecalciferol	92 (9.60)	27 (3.17)	65 (61.90)	< 0.0001
Native vitamin D^b^	71 (7.41)	60 (7.03)	11 (10.48)	0.2039
Calcitriol	18 (1.88)	12 (1.41)	6 (5.71)	0.0022
Alfacalcidol	16 (1.67)	11 (1.29)	5 (4.76)	0.0088
**Vitamin K2**	**122 (12.73)**	**121 (14.19)**	**1 (0.95)**	**0.0001**
Menaquinone	75 (7.83)	75 (8.79)	0 (0.00)	0.0016
Menatetrenone	47 (4.91)	46 (5.39)	1 (0.95)	0.0468
**Bisphosphonates**	**86 (8.98)**	**66 (7.74)**	**20 (19.05)**	**0.0001**
Alendronate sodium	38 (3.97)	28 (3.28)	10 (9.52)	0.0020
Zoledronic acid	30 (3.13)	21 (2.46)	9 (8.57)	0.0007
Ibandronate sodium	16 (1.67)	15 (1.76)	1 (0.95)	0.5430
**Denosumab**	**235 (24.53)**	**217 (25.44)**	**18 (17.14)**	**0.0623**
Other	**784 (81.84)**	**698 (81.83)**	**86 (81.90)**	**0.9848**

Bold text indicates medication classification categories

Non-bold text indicates individual medications within each classification category

All data presented are n (%). Percentages are calculated based on the total number of patients per cohort (N)

*Ca* calcium, *N* the number of enrolled patients, *n* the number of patients in each category

^a^Medications were classified as concomitant if their administration period overlapped by at least one day with the period from the first to the last dose of eldecalcitol treatment

^b^“Native vitamin D” refers to vitamin D reported without specification as to whether it contains vitamin D2, vitamin D3, or a combination of both

### Drug exposure

The mean (SD) initial daily dose of eldecalcitol in the study population was 0.75 (0.02) μg. The mean (SD) daily dose of eldecalcitol during the whole study period was 0.75 (0.04) μg. The median (min, max) exposure to eldecalcitol in the overall population was 354.0 days (1.0–478.0), which was shorter than the actual treatment period. Specifically, the without Ca group had a median (min, max) exposure of 353.0 days (1.0–478.0), while the with Ca group's median exposure was 357.0 days (3.0–476.0) (Table S1).

At the start of eldecalcitol treatment, calcium supplementation mainly comprised calcium carbonate with “calcium carbonate; cholecalciferol” and “calcium carbonate”, with mean (SD) doses of elemental calcium 677.27 (812.80) mg and 577.50 (156.00) mg, respectively. During treatment in the with Ca supplementation group, calcium supplementation mainly comprised calcium carbonate with “calcium carbonate; cholecalciferol” and “calcium carbonate”, with mean (SD) doses of elemental calcium 650.96 (804.34) mg and 615.91 (229.59) mg, respectively. A decreasing trend of Ca use was observed for those with Ca at baseline (Table 2B). For patients without Ca at baseline, less than 5% received Ca at 3, 6, 9, and 12 months.

### Primary outcome: incidence of hypercalcemia

Twenty-two patients (2.30%) in the overall group experienced hypercalcemia including 17 (1.99%) and 5 (4.76%) in the without and with Ca supplementation groups, respectively.

Although univariate logistic regression analysis did not reach statistical significance, results showed that the group with Ca was more than twice as likely to develop hypercalcemia versus the group without Ca (Table 4; n = 17; OR: 2.46; 95% CI 0.89, 6.81). Multivariable logistic regression analysis was performed to adjust for confounding factors such as status of Ca use at baseline, age, baseline eGFR, and primary hyperparathyroidism. It should be noted, however, that 258 patients did not have baseline eGFR values. The multivariable analysis indicated that the group with Ca was three times more likely to experience hypercalcemia versus the without Ca group (OR: 3.07; 95% CI 1.16, 8.10, *P* = 0.02). Patients with fragility fractures also had a greater risk of hypercalcemia (adjusted OR: 4.08; 95% CI 1.40, 11.91). In a post hoc analysis of the clinical characteristics of patients who developed hypercalcemia, there were no substantial differences in baseline renal function or baseline serum calcium levels between patients who developed hypercalcemia and those who did not (Table S3). Regarding concomitant medications, a higher proportion of patients who developed hypercalcemia received calcium supplements, and a significantly higher proportion received vitamin D analogs compared with those who did not develop hypercalcemia (Table S3). In addition, anti-receptor activator of nuclear factor kappa-Β ligand (RANKL) therapy (denosumab) was more frequently administered among patients who developed hypercalcemia. However, in the univariate analysis stratified by anti-RANKL (denosumab) use, the odds ratio was 1.137 with a 95% CI of 0.380–3.407, and no statistically significant association was observed (*P* = 0.8183). Therefore, no significant effect of anti-RANKL (denosumab) use on the development of hypercalcemia was identified. Furthermore, when anti-RANKL (denosumab) use was included as an explanatory variable in the multivariate analysis, it was not selected in the final model, indicating that anti-RANKL (denosumab) use was not an independent factor associated with hypercalcemia.

**Table 4:** Logistic regression on the association between hypercalcemia and the status of calcium use

	Statistics	Without Ca (N = 853)	With Ca (N = 105)
Descriptive	n (%)	17 (1.99)	5 (4.76)
	95% CI	(1.06, 2.93)	(0.69, 8.84)
Univariate logistic^a^	Crude odds ratio		2.46
	95% CI		(0.89, 6.81)
	*P*-value		0.08
Multivariable logistic^b^	Adjusted odds ratio		3.07
	95% CI		(1.16, 8.10)
	*P*-value		0.02

Percentages are calculated based on the total number of patients per cohort (N) in the safety analysis set

*Ca* calcium, *CI* confidence interval, *N* the number of patients in safety analysis set, *n* the number of patients in each category

^a^With only the status of Ca use

^b^With the status of Ca use adjusted for the selected prognostic variables based on the stepwise selection method

**Fig. 2:**
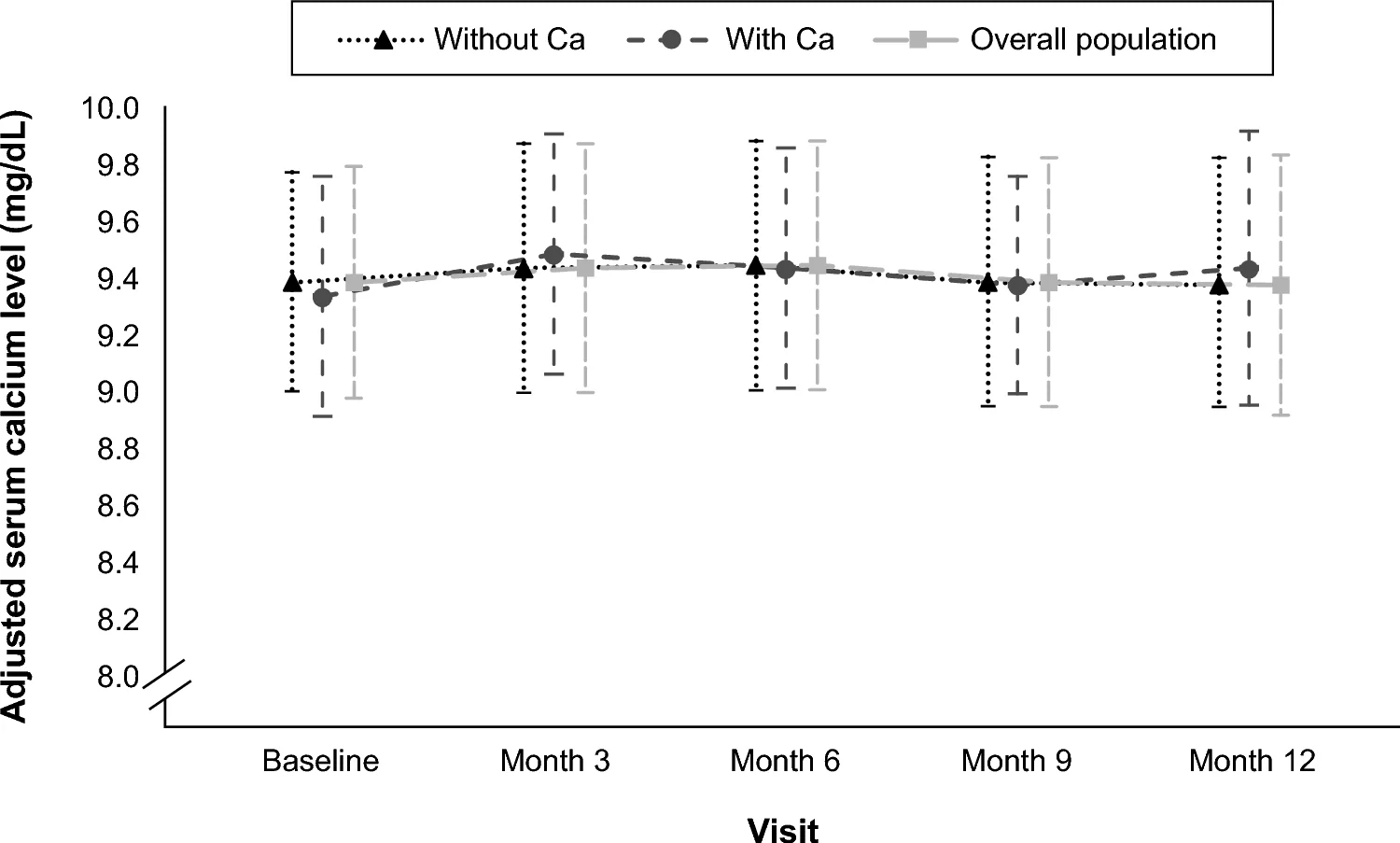
Mean adjusted serum calcium levels at 3, 6, 9, and 12 months. Data are means and SD. *Ca* calcium, *SD* standard deviation

In laboratory testing, the adjusted sCa levels from baseline to 12 months remained relatively stable over time (Fig. 2) with the results for the without Ca and with Ca groups being similar. The impact of eldecalcitol treatment on sCa > 10.4 mg/dL included 28 events in 28 patients (2.92%; Table S2). There was no statistically significant difference between the two groups (Table S2). For uCa > 300 mg/day on 2 consecutive time points, overall, three events related to eldecalcitol were reported in 3 (0.31%) patients; of these, 1 event in 1 patient (0.12%) was reported in the without Ca group, while 2 events in 2 patients (1.89%) were reported in the with Ca group.

The adjusted sCa levels from baseline to 12 months remained relatively stable over time (Fig. 2), with the results for the with Ca and without Ca groups being similar.

### Summary of AEs

AEs were experienced by 445 (52.17%) and 61 (58.10%) patients in the without and with Ca groups, respectively. Of these, 85 (9.96%) patients in the without Ca group and 16 (15.24%) patients in the with Ca group experienced SAEs (Table 5). No significant differences were observed between the without Ca group and the with Ca group with respect to AEs, SAEs, ADRs, or SADRs.

**Table 5:** Summary of adverse events during follow up

Incidence of AEs	Overall population (N = 958)	Without Ca (N = 853)	With Ca (N = 105)	*P*-value
General				
Any AEs	506 (52.82%)	445 (52.17%)	61 (58.10%)	0.2510
Any ADRs	379 (39.56%)	334 (39.16%)	45 (42.86%)	0.4642
Any SAEs	101 (10.54%)	85 (9.96%)	16 (15.24%)	0.0969
Any SADRs	56 (5.85%)	52 (6.10%)	4 (3.81%)	0.3460
Discontinued due to AEs	38 (3.97%)	32 (3.75%)	6 (5.71%)	0.3308
Discontinued due to ADRs	38 (3.97%)	32 (3.75%)	6 (5.71%)	0.3308
Death	1 (0.10%)	1 (0.12%)	0 (0.00%)	0.7256
Hypercalcemia ADRs^a^	22 (2.30%)	17 (1.99%)	5 (4.76%)	0.0739
Urolithiasis ADRs^b^	3 (0.31%)	3 (0.35%)	0 (0.00%)	0.5428
ADRs, other than hypercalcemia, with incidences > 2% in patients				
Upper respiratory tract infection	42 (4.38%)	37 (4.34%)	5 (4.76%)	0.8412
Urinary tract infection	21 (2.19%)	19 (2.23%)	2 (1.90%)	0.8313
Blood 25-hydroxycholecalciferol decreased	21 (2.19%)	21 (2.46%)	0 (0.00%)	0.1040
Constipation	20 (2.09%)	19 (2.23%)	1(0.95%)	0.3885
Abdominal discomfort	15 (1.57%)	12 (1.41%)	3 (2.86%)	0.2586
Arthralgia	14 (1.46%)	10 (1.17%)	4 (3.81%)	0.0336
Respiratory tract infection	12 (1.25%)	9 (1.06%)	3 (2.86%)	0.1172
Urine calcium increased	12 (1.25%)	9 (1.06%)	3 (2.86%)	0.1172
Pruritus	8 (0.84%)	5 (0.59%)	3 (2.86%)	0.0158
Spinal osteoarthritis	7 (0.73%)	4 (0.47%)	3 (2.86%)	0.0067
Rash	6 (0.63%)	3 (0.35%)	3 (2.86%)	0.0021
Pulmonary mass	5 (0.52%)	2 (0.23%)	3 (2.86%)	0.0004
Rib fracture	5 (0.52%)	2 (0.23%)	3 (2.86%)	0.0004
Hypercalciuria	4 (0.42%)	1 (0.12%)	3 (2.86%)	< 0.0001

Data are compiled using ICH Medical Terminology MedDRA Ver 27.1. The AE descriptions in the table are PTs

All data are reported as n, number of patients (%). Percentages are calculated based on the total number of patients per cohort (N)

*ADR* adverse drug reaction, *AE* adverse event, *Ca* calcium, *N* the number of patients in safety analysis set, *n* the number of patients in each category, *PT* Preferred Term, *SAE* serious adverse event; SADR, serious adverse drug reaction

^a^Includes “hypercalcemia” or “blood calcium increased”

^b^Defined as AEs whose PT is “ureterolithiasis”, “nephrolithiasis” or “urinary calculus”

ADRs were reported by 334 (39.16%) and 45 (42.86%) patients in the without and with Ca groups, respectively. Serious ADRs were reported by 52 (6.10%) and 4 (3.81%) patients in the without and with Ca groups. Serious ADRs that were reported in the without Ca group included cerebral infarction (5 events in 5 patients, 0.59%), diabetic complications (4 events in 4 patients, 0.47%), and pneumonia (3 events in 3 patients, 0.35%). In the with Ca group the serious ADRs pneumonia, diabetic neuropathy, transient ischemic attack, and anti-neutrophil cytoplasmic antibody positive vasculitis occurred once in individual patients (0.95%). For ADRs other than hypercalcemia with incidences > 2% in patients, arthralgia, pruritus, spinal osteoarthritis, rash, pulmonary mass, rib fracture, and hypercalciuria were present in a significantly higher proportion of patients in the with Ca group than in the without Ca group. Thirty-two (3.75%) and 6 (5.71%) patients discontinued treatment due to ADRs in the without and with Ca groups, respectively. There was 1 death (0.12%) in the without Ca group, which was the result of a cerebrovascular disorder possibly unrelated to study treatment. There were 3 ADRs of urolithiasis in 3 patients (0.35%) in the without Ca group and no cases in the with Ca group. Two patients (0.23%) reported non-serious urolithiasis, resulting in drug discontinuation for 1 patient and not applicable for the other as the patient had discontinued eldecalcitol on the day before the event. A serious ADR of urolithiasis was also reported in 1 patient (0.12%), who recovered following drug discontinuation. No cases of acute kidney injury were reported.

### Hypercalcemia event outcomes and management

Among the 22 patients with hypercalcemia related to eldecalcitol, 7 continued administration, 3 had their dose reduced, 9 had their dose interrupted/discontinued, for 2 patients the action was described as not applicable, and 1 patient took other action (continued administration then temporarily suspended). In the without Ca group (17/853 patients), the events were reported as recovered in 12 patients, and in the with Ca group (5/105 patients), the events recovered in 1 patient. At the time of data cut-off, no hypercalcemia event was reported as recovered with sequelae. There were no hypercalcemia-related deaths in either group.

### Hypercalcemia in patients without hyperparathyroidism

Ten patients with primary hyperparathyroidism were enrolled. Therefore, an additional analysis was conducted to exclude patients with primary hyperparathyroidism and explore the impact of Ca co-administration on hypercalcemia in the population without primary hyperparathyroidism. Even when excluding patients with primary hyperparathyroidism, multivariable logistic regression analysis indicated that the impact of Ca on hypercalcemia remained high, exhibiting a higher risk (OR: 3.25; 95% CI 1.23, 8.58, *P* = 0.02) for developing hypercalcemia.

## Discussion

This observational cohort study conducted in Chinese women with postmenopausal osteoporosis reported an increased risk for hypercalcemia with eldecalcitol and concomitant Ca supplementation.

In this study, groups were divided based on calcium supplementation status at baseline. The calcium supplementation status changed during the follow-up period, which could have been caused by physician judgment or patient preferences. Even under such situations, throughout the entire observation period, the proportion of patients who continuously received calcium supplementation in the with Ca group was higher than the proportion of patients who received calcium supplementation during the observation period in the without Ca group. Therefore, grouping based on whether calcium supplementation was initially administered and conducting subsequent statistical analyses are considered to adequately estimate the impact of the presence or absence of calcium supplementation on the safety of eldecalcitol. Excluding calcium supplements, the medications that were used more frequently in the with Ca group were vitamin D and vitamin D analogs, as well as bisphosphonates, while vitamin K2 was used more frequently in the without Ca group. While the impact of all comorbidities on the occurrence of hypercalcemia cannot be fully accounted for, a multivariable analysis was conducted to control for potential confounding variables, using pre-specified factors considered to have a potential influence on the development of hypercalcemia at the initiation of eldecalcitol treatment as explanatory variables. Based on this analysis, it was possible to confirm a higher incidence of hypercalcemia in the with Ca group.

The safety results in this drug intensive monitoring study, including incidence of hypercalcemia, acute kidney injury, and urolithiasis, were aligned with the safety profile of eldecalcitol seen in clinical trials and post-marketing studies to date. The incidence of hypercalcemia ADRs in the present study (2.30%, 1.99%, and 4.76% in the overall population, without Ca group, and with Ca group, respectively) was comparable with the known safety data of eldecalcitol. In a Chinese phase 3 clinical trial, 5.9% of patients experienced an AE of hypercalcemia (sCa > 10.4 mg/dL) and 0% experienced sCa > 11.0 mg/dL [9]. Similarly, in a post-marketing surveillance study in Japan, 0.94% of women experienced hypercalcemia or increased blood calcium ADRs [11].

While the impact of all comorbidities on the occurrence of hypercalcemia cannot be fully accounted for, a multivariable analysis was conducted with explanatory variables of factors considered to have a potential influence on the development of hypercalcemia at the initiation of eldecalcitol treatment. Based on this analysis, it was possible to confirm a higher incidence of hypercalcemia in the with Ca group.

In line with the Japanese and Chinese phase 3 clinical trials [8, 9] and the post-marketing surveillance study in Japan [11], there were no reports of acute kidney injury in this observational cohort study. Urolithiasis is one of the safety concerns with eldecalcitol. In a Japanese phase 3 clinical trial, seven patients (1.32%) experienced an AE of urolithiasis [8], and one patient (0.03%) experienced a serious ADR of nephrolithiasis in the Japanese post-marketing surveillance [11]. There were 3 cases (0.31%) of urolithiasis in the current study, which all occurred in the without Ca group. Based on the current study, no new safety concerns regarding urolithiasis emerged in this Chinese real-world setting.

There was no notably higher incidence of ADRs compared with the expected levels per the known safety profile of eldecalcitol [8, 9, 12]. In the current study, AEs were reported by 52.17% and 58.10% of patients in the without and with Ca groups, respectively, over 12 months of follow-up in this study. This compares with 98.50% of patients who reported AE in the Japanese phase 3 trial (NCT00144456), although that trial was over 3 years [8]. The number of patients impacted by AEs in the current study was also lower than observed in a 12-month Chinese phase 3 trial (JAPIC CTI 152904), in which 83.80% of patients experienced AEs with eldecalcitol [9]. In total, ADRs were reported by 379 patients (39.56%), which is higher than the 107 women (3.74%) of who experienced ADRs in the Japanese post-marketing surveillance study [11]. In the Japanese study, 6.76% of women were receiving Ca supplementation compared with 15.14% in the current study. In the current study, arthralgia, pruritus, spinal osteoarthritis, rash, pulmonary mass, rib fracture, and hypercalciuria occurred more frequently in the with Ca group than in the without Ca group.

A serious ADR to note was cerebral infarction, which was reported in 5 patients (0.59%) in the without Ca group, whereas no cases were reported in the with Ca group. There is no evidence to confirm that eldecalcitol increases the risk for cerebral infarction. A higher incidence of this AE was reported in the Chinese study and the current study compared to those conducted in Japan (data on file), likely because of differences in epidemiological data between the 2 countries. For example, in Japan, the estimated incidence of ischemic stroke (including both first-ever and recurrent events) among women is 125.0 per 100,000 person-years [14]. In contrast, in China, the estimated incidence of ischemic stroke (including both first-ever and recurrent events) among women is 213.4 per 100,000 person-years [15].

While the study aligns with prior findings on Ca and vitamin D co-supplementation, our study used eldecalcitol, a vitamin D analog with greater calcemic activity than native vitamin D, highlighting the need for caution when combining Ca with active vitamin D analogs [13].

### Limitations

While offering valuable insights for comparable populations and settings, the study was also subject to limitations common to real-world observational study designs. For example, site selection was not randomized, so enrollment may have been unbalanced across study sites. AEs and Medical history were reported according to local clinical practice, which may have resulted in some inconsistencies. Due to the real-world approach, patients may have missed some of their clinical visits, resulting in incomplete laboratory values and potentially underestimating the effect of concomitant Ca use on hypercalcemia. Secondly, follow-up frequency was non-mandatory, relying on routine clinical visits within an allowed window rather than a strict protocol. Lastly, study limitations include that the multivariable model was built including patients with eGFR < 60 mL/min/1.73 m^2^; however, there were 258 patients for whom eGFR values were missing. This missing data may have resulted in inadequate adjustment for confounding.

### Conclusions

This post-marketing surveillance study confirmed the consistent safety profile of eldecalcitol in postmenopausal women with osteoporosis in China. The incidence of ADRs, including hypercalcemia and urolithiasis, was similar to previous data. Hypercalcemia was higher in patients receiving Ca versus those without Ca supplementation. These findings highlight the need to evaluate the necessity of concomitant Ca use with eldecalcitol treatment and provide valuable real-world evidence to help guide the clinical management of postmenopausal women with osteoporosis.

## Supplementary Information

Below is the link to the electronic supplementary material.

Supplementary file1 (DOCX 18 kb)

## Data Availability

The datasets generated during and/or analyzed during the current study are not publicly available. Because of the observational nature of this research, participants of this study did not agree for their data to be shared publicly.
