# Supplementary material for: Eldecalcitol and calcium supplementation in Chinese women with postmenopausal osteoporosis: a prospective observational cohort study
Source: J Bone Miner Metab. 2026 Jun 16;44(5):597–609. doi: 10.1007/s00774-026-01735-x (PMC13723487; doi:10.1007/s00774-026-01735-x)
Supplement: Supplementary file 1 [file 774_2026_1735_MOESM1_ESM.docx]

## **Supplementary material**

### **Supplementary Table S1.** Exposure to the study drug

| **Treatment exposure** |  | **Overall  population (N=958)** | **Without Ca  (N=853)** | **With Ca  (N=105)** |
| --- | --- | --- | --- | --- |
| **Mean duration of actual treatment period (days) [1]**  **Median**  **Min–max** |  | 289.16±133.74  354.00  1.00–478.00 | 291.29±131.91  353.00  1.00–478.00 | 271.86±147.27  357.00  3.00–476.00 |
| **Mean total number of days exposed to study drug (days) [2]**  **Median**  **Min–max** |  | 269.49±136.56  337.00  1.00–478.00 | 272.26±135.45  339.00  1.00–478.00 | 247.06±144.00  282.00  3.00–476.00 |
| **Mean total doses taken (μg) [3]**  **Median**  **Min–max** |  | 201.28±102.93  (n=946)  252.75  0.75–358.50 | 203.26±102.18  (n=843)  253.50  0.75–358.50 | 185.11±108.03  (n=103)  211.50  2.25–357.00 |

Data presented are mean ± SD, unless otherwise stated.

[1] Duration of actual treatment period (days) = (the last date of study drug – the first date of study drug + 1).

[2] Total number of days exposed to study drug (days) = sum of all records of (the last date of study drug – the start date of study drug + 1).

[3] Total doses taken (μg) = sum of all records of duration (the last date of study drug – the start date of study drug + 1) × frequency × dose.

**Ca**, calcium; **N**, the number of patients in safety analysis set; **n**, the number of patients in each category; **SD**, standard deviation.

### **Supplementary Table S2.** Logistic regression on the association between adjusted sCa >10.4 mg/dL and the status of Ca use (SAS)

| **Item** | **Statistics** | **Without Ca (N=853)** | **With Ca (N=105)** |
| --- | --- | --- | --- |
| Descriptive | n (%) | 26 (3.05) | 2 (1.90) |
|  | 95% CI | (1.89, 4.20) [a] | (0.23, 6.71) [b] |
| Univariate Logistic [1] | Crude odds ratio |  | 0.618 |
|  | 95% CI |  | (0.144, 2.640) |
|  | *P*-value |  | 0.5156 |
| Multivariable Logistic [2] | Adjusted odds ratio |  | 0.751 |
|  | 95% CI |  | (0.210, 2.681) |
|  | *P*-value |  | 0.6589 |

**N**, the number of patients in SAS; **n**, the number of patients in category.

Percentage (%) = 100 * (n / N).

[a] Wald CI; [b] Clopper-Pearson CI.

[1] With only the status of Ca use.

[2] With the status of Ca use adjusted for the selected prognostic variables based on the stepwise selection method.

**Ca**, calcium; **CI**, confidence interval; **SAS**, safety analysis set; **sCa**, serum calcium.

### **Supplementary Table S3.** Clinical characteristics of patients who did and did not development hypercalcemia.

| **Item** | **Classification and/or statistics** | **No hypercalcemia (including blood calcium increased)** | **Hypercalcemia (including blood calcium increased)** | **Total** | **OR (95% CI)** | ***P*-value** |
| --- | --- | --- | --- | --- | --- | --- |
| Baseline adjusted sCa  (mg/dL) [1] | Nx | 790 | 16 | 806 |  |  |
|  | Mean±SD | 9.38±0.40 | 9.44±0.50 | 9.39±0.41 |  |  |
|  | Median | 9.36 | 9.5 | 9.36 |  |  |
|  | [Min–Max] | [7.88–11.32] | [8.40–10.32] | [7.88–11.32] |  |  |
|  | Nmiss | 146 | 6 | 152 |  |  |
|  | >10.4 [2] | 5 (0.63) | 0 (0.00) | 5 (0.62) | <0.001 (<0.001, >999.999) | 0.9921 |
| Baseline eGFR  (mL/min/1.73m^2^) [3] | Nx | 689 | 11 | 700 |  |  |
|  | Mean±SD | 90.32±15.33 | 88.10±11.67 | 90.28±15.27 |  |  |
|  | Median | 93.6 | 92.6 | 93.6 |  |  |
|  | [Min-Max] | [24.1–119.3] | [60.8–101.0] | [24.1–119.3] |  |  |
|  | Nmiss | 247 | 11 | 258 |  |  |
|  | <30, n (%) | 3 (0.44) | 0 (0.00) | 3 (0.43) |  |  |
|  | 30-44, n (%) | 5 (0.73) | 0 (0.00) | 5 (0.71) |  |  |
|  | 45-59, n (%) | 23 (3.34) | 0 (0.00) | 23 (3.29) |  |  |
|  | 60-89, n (%) | 233 (33.82) | 5 (45.45) | 238 (34.00) |  |  |
|  | ≥90, n (%) | 425 (61.68) | 6 (54.55) | 431 (61.57) |  |  |
|  | <60 [2] | 31 (4.50) | 0 (0.00) | 31 (4.43) | 0.909 (0.050, 16.482) | 0.9485 |
| Calcium | n (%) | 139 (14.85) | 6 (27.27) | 145 (15.14) | 2.151 (0.827, 5.590) | 0.1162 |
| Native vitamin D | n (%) | 69 (7.37) | 2 (0.09) | 71 (7.41) | 1.257 (0.288, 5.488) | 0.7612 |
| Vitamin D analogs (Calcitriol, Alfacalcidol) | n (%) | 30 (3.21) | 3 (13.64) | 33 (3.44) | 4.769 (1.338, 16.994) | 0.0160 |
| Bisphosphonates | n (%) | 83 (8.87) | 3 (13.64) | 86 (8.98) | 1.623 (0.470, 5.598) | 0.4435 |
| Vitamin K2 | n (%) | 121 (12.93) | 1 (4.55) | 122 (12.73) | 0.321 (0.043, 2.406) | 0.2687 |
| Denosumab | n (%) | 227 (24.25) | 8 (36.36) | 235 (24.53) | 1.785 (0.739, 4.309) | 0.1977 |
| Teriparatide | n (%) | 18 (1.92) | 0 (0.00) | 18 (1.88) | 1.103 (0.060, 20.382) | 0.9476 |

[1] sCa + (4 – Albumin) if Albumin <4.0 g/dL and sCa if Albumin ≥4.0 g/dL.

[2] Unknown category for the Nmiss was included in the logistic regression analysis.

[3] 141 × minimum (sCr/0.7, 1) ^(-0.329)^ × maximum (sCr/0.7, 1) ^(-1.209)^ × 0.993 ^Age^ × 1.018.

**CI**, confidence interval; **eGFR**, estimated glomerular filtration rate; **Max**, maximum; **Min**, minimum; **Nmiss**, the number of patients with missing data for each variable; **Nx**, the number of patients with non-missing data; **OR**, odds ratio; **sCa**, serum calcium; **sCr**, serum creatinine; **SD**, standard deviation**.**
